# Meningitis Retention Syndrome With Mild Encephalopathy With a Reversible Splenial Lesion in a 30-Year-Old Woman: A Case Report

**DOI:** 10.1155/crra/7331226

**Published:** 2025-03-13

**Authors:** Takuma Usuzaki, Tadayoshi Kato, Yohei Morishita, Hiroaki Furukawa, Kazuhiro Majima

**Affiliations:** ^1^Department of Diagnostic Radiology, Tohoku University Hospital, Sendai, Japan; ^2^Department of Neurology, Takeda General Hospital, Aizuwakamatsu, Japan; ^3^Department of Radiology, Takeda General Hospital, Aizuwakamatsu, Japan

**Keywords:** meningitis, meningitis retention syndrome, mild encephalitis/encephalopathy with a reversible splenial lesion, retention

## Abstract

We describe a 30-year-old woman who had meningitis retention syndrome (MRS) with mild encephalitis/encephalopathy with a reversible splenial lesion (MERS), which occurred with fever, urinary retention, and weakness in both legs. A case of MRS with MERS is rare among adults, and its clinical course and treatment planning remain unknown. In the present, we highlighted the change in magnetic resonance imaging, blood tests, and cerebrospinal tests along with the treatment. A multidisciplinary approach by a radiologist and neurologist led to the diagnosis and appropriate treatment.

## 1. Introduction

Meningitis retention syndrome (MRS) is defined as coexisting aseptic meningitis and acute urinary retention in the absence of any other disease that might cause urinary retention [1]. After its first report by Sakakibara et al. in 2005 [2], MRS has been associated with viruses [3, 4], bacteria [5], drugs [6], and other diseases [7]. Although the pathophysiology of MRS is not yet understood, it has been speculated that viral/bacterial inflammation or postinfection inflammatory demyelination can cause sacral myeloradiculopathy [8]. Pellegrino et al. reviewed 29 cases of MRS [9]. They reported that the age at diagnosis ranged from 13 to 74 years, and male to female ratio was 2.3. The rate of occurrence of MRS in those with aseptic meningitis has been reported as 8% [10]. Since the incidence of aseptic meningitis is 7.5 cases per 100,000 in a year [11], the incidence of MRS can be estimated at 0.6 cases per 100,000 in a year.

A young adult man of MRS reported by Hidaka et al. simultaneously exhibited mild encephalitis/encephalopathy with a reversible splenial lesion (MERS) [8]. Hidala et al. suggested that severe MRS may exhibit refractory urinary retention associated with MERS. However, MERS in adults is less common than in children [12], and there is a difficulty in the accumulation of adult cases with MRS and MERS. Takanashi reviewed 54 Japanese patients with MERS [12]. This study revealed that the mean age at onset was 9 years, and male to female ratio was 0.93 [13]. Therefore, it is necessary to perform a detailed investigation of the clinical course in an adult patient with MRS with MERS. This report describes a case of MRS with MERS, including its treatment and image findings, in a 30-year-old woman.

## 2. Case Presentation

A 30-year-old woman presented to the emergency department with fever, urinary retention, and weakness in both legs. She had developed a temperature of up to 39.0°C and headache 1 week prior and urinary retention 2 days prior to presentation. On examination, her temperature was 38.3°C, blood pressure was 133/97 mmHg, heart rate was 100 beats per minute, and oxygen saturation was 100% while breathing ambient air. The distension of the lower abdomen was caused by retention, and 1100 mL of urine was drained by temporary catheterization. The patient had a history of coronavirus disease 2019 (COVID-19) and candida vaginitis. The treatment of COVID-19 was terminated 3 months ago, and candida vaginitis was under treatment. Her current medications included drospirenone and levocetirizine hydrochloride. Neurological examination by a neurologist revealed stiff neck, jolt-accentuation sign, Kernig sign, mild hyperalgesia in the area governed by the 10th–11th thoracic nerve and lumbar nerves, brisk hyperreflexia of both sides, and pseudoclonus of the right leg. Decreased level of consciousness, seizures, and irritability were not observed. Her white blood cell (WBC) count was 12,800/*μ*L (reference range, 3600–8100), and C-reactive protein (CRP) was 0.21 mg/dL (0.00–0.14). Analysis of cerebrospinal fluid (CSF) revealed a cell count of 43 (0–15), protein of 190 mg/dL (10–40), glucose of 26 mg/dL (40–70), and interleukin (IL)-6 of 5.6 pg/mL (0.0–4.0). Candida antigen and *β*-D-glucan were not detected in CSF. Both blood and CSF cultures were negative. Herpes simplex virus and varicella zoster virus were not detected in CSF using polymerase chain reaction.

### 2.1. Image Findings at Hospitalization and Follow-Up

**Figure 1:**
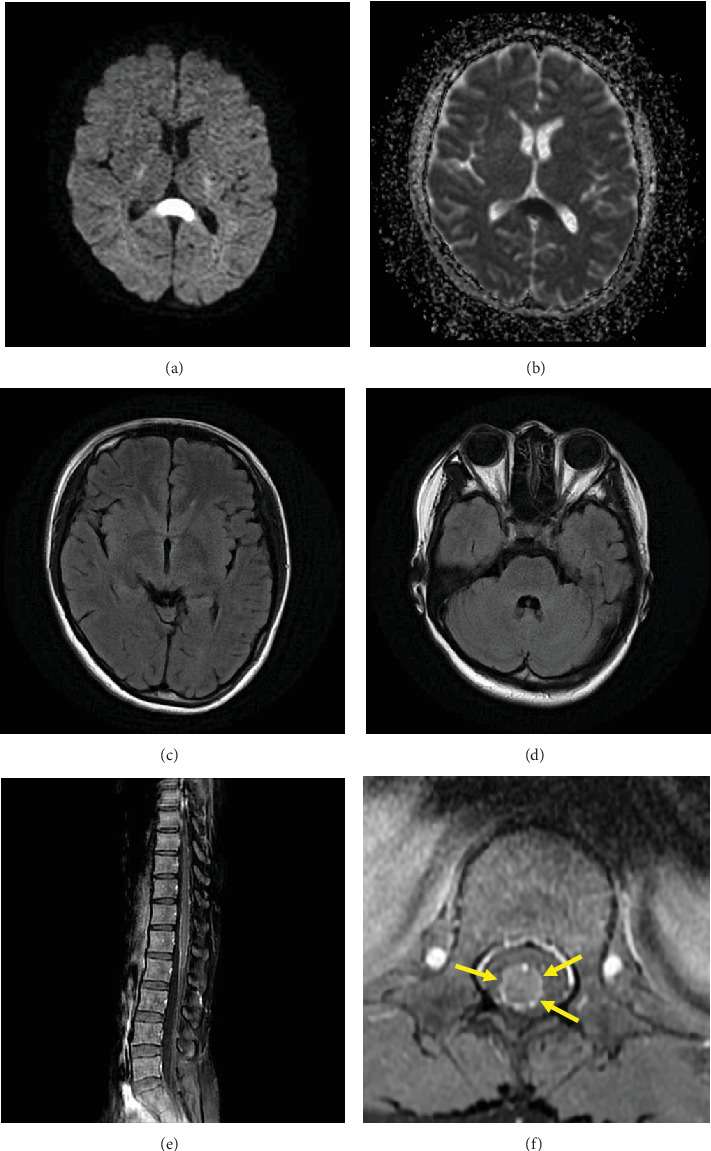
Magnetic resonance imaging (MRI) at hospitalization. Diffusion-weighted imaging (DWI) showed high signal intensity in the splenium of the corpus callosum (a). Signal reduction in the same area was observed on the ADC map (b). FLAIR images did not show any abnormal signal areas other than the splenium of the corpus callosum (c, d). Sagittal and axial T1-weighted contrast-enhanced images of the lower spine (e, f) showed contrast enhancement on the surface of conus medullaris (f, arrows) and expansion of the surrounding vessels.

**Figure 2:**
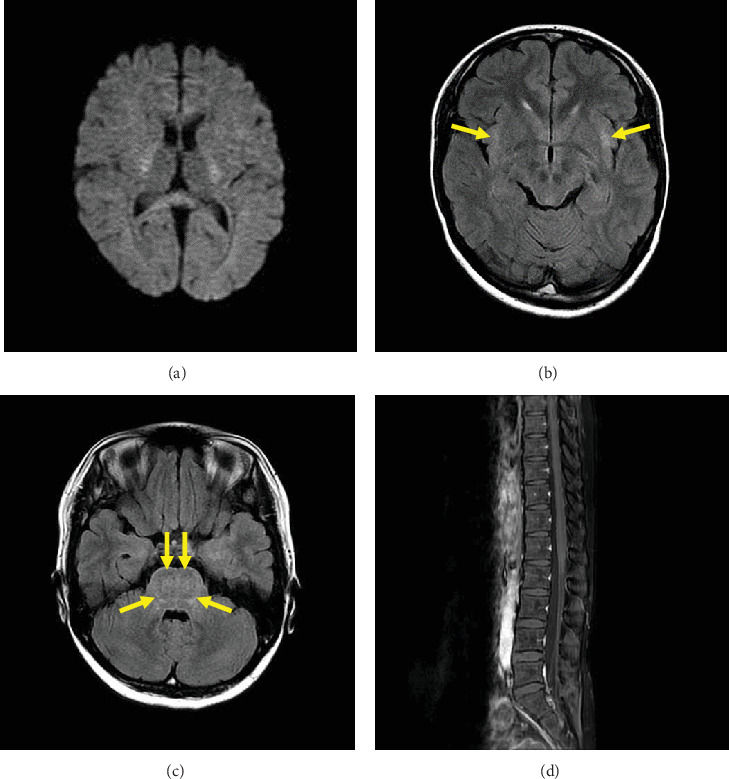
Magnetic resonance imaging (MRI) on Day 14. DWI showed reduced signal intensity in the SCC (a). FLAIR images revealed high signal intensity in the external capsule (b, arrows) and pons (c, arrows). Sagittal T1-weighted contrast-enhanced images of the lower spine showed no abnormal enhancement (d).

Head computed tomography (CT) showed no abnormalities, and abdominal and pelvic CT showed no other cause of urinary retention. Noncontrast magnetic resonance imaging (MRI) of the brain at hospitalization revealed high signal intensity in the splenium of the corpus callosum (SCC) on diffusion-weighted imaging (DWI) (Figure 1a). Signal reduction in the same area was observed on the ADC map (Figure 1b). FLAIR images did not reveal any abnormal signal areas other than the SCC (Figure 1c,d). Contrast-enhanced MRI of the lower spine revealed contrast enhancement on the pia mater of conus medullaris (Figure 1e,f). Ischemic infarction, acute disseminated encephalomyelitis (ADEM), multiple sclerosis (MS), Marchiafava–Bignami disease (MBD), osmotic demyelinating syndrome (ODS), and autoimmune glial fibrillary acidic protein (GFAP) astrocytopathy were excluded by image findings [13, 14]. Twenty-four days after hospitalization, a brain noncontrast MRI was repeated. DWI showed reduced signal intensity in the SCC (Figure 2a). FLAIR images revealed high signal intensity in the external capsule (Figure 2b, arrows) and pons (Figure 2c, arrows), which may suggest postinfectious encephalopathy. The previously observed strong contrast enhancement on the pia mater of the conus medullaris on the lower spine MRI had disappeared by Day 32.

### 2.2. Diagnosis and Treatment

Based on the neurological findings, symptoms of infection, and blood test, she had meningitis. She was diagnosed with aseptic meningitis because any bacteria and fungus were not detected for blood and CSF. The mild elevation of the cell counts and protein level partly supported this diagnosis. In addition to retention and aseptic meningitis, the patient had mild encephalitis/encephalopathy symptoms and a reversible splenial lesion on MRI. In short, she simultaneously had retention, aseptic meningitis, and MERS. Thus, the patient was diagnosed with MRS with MERS.

**Figure 3:**
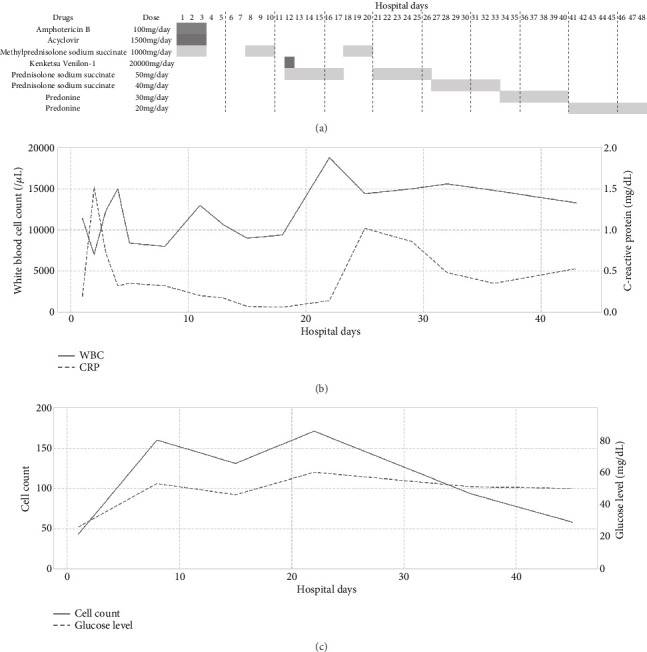
Treatment, blood test, and cerebrospinal fluid (CSF) test results. (a) Drugs used to treat the patient were summarized. (b) Changes in white blood cell count (left vertical axis) and C-reactive protein (right vertical axis) are shown. (c) Changes in cell count (left vertical axis) and CSF glucose level (right vertical axis) are shown. The horizontal axis shows hospital days in both (b) and (c).

Urinary drainage was performed after admission to the hospital, and glucocorticoid steroid, acyclovir, and amphotericin B were started. When we started administering amphotericin B, the results of the CSF test were unknown. We administered amphotericin B because there was the possibility of fungus meningitis. Amphotericin B was terminated after checking the candida antigen, and *β*-D-glucan in CSF was negative. The patient's temperature, meningeal irritation, brisk hyperreflexia, pseudoclonus, and hyperalgesia had improved by hospitalization Day 2. Twenty-four days after hospitalization, urinary retention improved, and urinary drainage was terminated.  Figure 3a summarizes the treatment schedule.  Figure 3b,c shows the change in blood test (WBC and CRP) and CSF test (cell count and glucose level) during hospitalization, respectively. Twenty-four days after hospitalization, the recurrence of aseptic meningitis was suspected from the elevation of WBC, CRP, and cell count. On the other hand, most symptoms and image findings improved. We determined to continue steroid administration for two reasons: first, the symptoms improved after steroid administration, and second, autoimmune encephalitis could not be entirely excluded [15]. The patient was discharged 48 days after admission because the symptoms disappeared and laboratory data got better. After discharge, the patient was prescribed 10 mg of predonine per day, which was tapered over 4 weeks and then terminated. During the 6-month follow-up after discharge, there were no symptoms of recurrence.

## 3. Discussion

In the present paper, we describe the case of a 30-year-old woman who had MRS with MERS, which, to the best of our knowledge, is the first report in a young adult woman. The incidence of aseptic meningitis in a year is estimated at 7.5 cases per 100,000 among adults and is three times more common in males than females [11]. The MRS occurred in 8% of patients with aseptic meningitis [10]. The precise incidence rate of MERS has not been reported. However, MERS is not common among adults [16, 17]. The present case report is novel in presenting the clinical course, treatment, and image findings to young adult women with a combination of MRS and MERS. In this case, a multidiscipline approach by radiologists and neurologists played an essential role in diagnosis and treatment.

MRS is a peculiar combination of acute urinary retention and aseptic meningitis [2]. Although patients with MRS are generally admitted to the hospital for treatment of urinary retention, the retention usually improves within 1–2 weeks without specific treatment [10]. The usual causes of acute urinary retention in adults include prostatic hyperplasia, peripheral nerve diseases involving the sacral spinal cord such as diabetic neuropathy and Guillain–Barré syndrome, and diseases of the lumbar spinal canal such as lumbar spondylosis and lumbar disc herniation [4]. These diseases should be considered and excluded to diagnose MRS. Although reports on spinal contrast-enhanced MRI findings in MRS are limited, many describe them as normal [4, 18]. On the other hand, a few reports have noted contrast enhancement of the pia mater of the conus medullaris, which was also observed in the present case [19, 20]. The period of urinary drainage in the present case was 24 days, which is slightly longer than previously reported for MRS [9]. The improvement in urinary retention was observed before the normalization of the CSF cell count. According to a previous report, other symptoms associated with MRS include meningeal irritation, brisk hyperreflexia, and pseudoclonus, as observed in the present case [10]. The effectiveness of immune treatments (e.g., steroid pulse therapy) for MRS remains unclear [21]. The present patient received glucocorticoid steroid, acyclovir, and amphotericin B. In a review of 28 MRS patients by Pellegrino et al. [9], the number of patients treated with steroids, antiviral drugs, and antifungal drugs was 3, 14, and 1, respectively [2–4, 22].

MERS is a parainfectious disorder that is apparent on MRI as a reversible SCC lesion that resolves between 3 days and 2 months [16, 23]. The SCC lesion observed in the present case disappeared after 24 days after hospitalization. The hypersensitivity seen in the present case can be explained by MERS [16]. MERS should be radiologically differentiated from ischemic infarction, ADEM, MS, MBD, ODS, and GFAP astrocytopathy [13, 14]. Based on clinical features, MERS can be classically considered a different form of ADEM [23]. MERS is regarded as localized cytotoxic edema in the SCC, which is rich in myelinated fibers, with a generally reversible and favorable prognosis. No encephalitic symptoms or signs are reported in most patients with MERS. On the other hand, ADEM often occurs after vaccination or exanthematous infections, and serological and pathological studies have suggested that it is a demyelinating condition of parainfectious or autoimmune origin [24]. ADEM typically presents on MRI with multiple bilateral asymmetrical subcortical white matter lesions, and this white matter damage may be permanent [23].

The association between MRS and MERS remains unknown [8, 9, 19]. Hidaka et al. [8] reported a 32-year-old man with MRS and MERS. In this case, urinary retention was refractory for 6 months after the SCC lesion disappeared on the 11th hospitalization day. In general, patients with MERS recovered completely within a month. This clinical course indicates that MERS may not be associated with urinary retention. Wang et al. [19] reported a 10-year-old girl with MRS and MERS. In this paper, the authors suggested that urinary retention is caused by localized inflammation in the meninges of the lower spinal cord. In this case, sagittal spinal MRI showed meningeal thickening and homogenous leptomeningeal enhancement of the thecal sac surrounding the conus medullaris and cauda equina on T1WI. The urination is controlled by pons and sacral spinal cords, and SCC is not associated with urination. We had the same position as these previous papers and diagnosed the present case as MRS with MERS. There is a slim chance that MERS explains urinary retention or one disease explains the pathology of the present case. MRS is defined as coexisting aseptic meningitis and acute urinary retention in the absence of any other disease that might cause urinary retention [1]. When urinary retention is explained by MERS, we may not be able to diagnose this case as MRS. However, the mechanism that causes MERS supports our diagnosis because MERS is the localized cytotoxic edema in the SCC with mild encephalopathy, and the association between MERS and urinary retention has not been directly confirmed. When diagnosing MRS, common diseases cause urinary retention, as mentioned above, and clinical courses should be carefully considered. The IL-6 elevation in CSF observed in the present and previous cases may support the hypothesis that strong cytokine releases due to MRS trigger MERS [8]. In addition to the MRI findings, it may be important to examine cytokine levels in CSF in patients with MRS. Although a previous study suggested that patients with MRS and MERS may have severe urinary symptoms, the patient in this paper had no recurrence of retention after urinary drainage was terminated [8]. Future studies should examine the effect of these therapies on disease and hospitalization periods.

## 4. Conclusion

We reported a case of MRS with MERS in a woman aged 30 years, which is much younger than previously reported cases. When MRS presents along with MERS, a radiologist may be consulted to make a diagnosis due to its characteristic image findings. In the present case, the neurologist first examined the patient, and the diagnosis was made by combining neurological with image findings. Although MRS and MERS are benign, severe MRS may exhibit refractory urinary retention associated with MERS. It is, therefore, important to adopt a multidisciplinary approach and perform a detailed investigation of the clinical course to ensure the proper treatment of these patients.

## Data Availability

We did not obtain consent to share the data from the patient, so due to the nature of the research, supporting data is not available.
